# Public Response to Federal Electronic Cigarette Regulations Analyzed Using Social Media Data Through Natural Language Processing: Topic Modeling Study

**DOI:** 10.2196/58919

**Published:** 2024-10-01

**Authors:** Shuo-Yu Lin, Sahithi Kiran Tulabandu, J Randy Koch, Rashelle Hayes, Andrew Barnes, Hemant Purohit, Songqing Chen, Bo Han, Hong Xue

**Affiliations:** 1 Department of Health Administration and Policy George Mason University Fairfax, VA United States; 2 WVU Health Affairs Institute Morgantown, WV United States; 3 Department of Psychology Virginia Commonwealth University Richmond, VA United States; 4 Department of Psychiatry Virginia Commonwealth University Richmond, VA United States; 5 Department of Health Behavior and Policy Virginia Commonwealth University Richmond, VA United States; 6 Department of Information Sciences and Technology George Mason University Fairfax, VA United States; 7 Department of Computer Science George Mason University Fairfax, VA United States

**Keywords:** social media analysis, data mining, natural language processing, topic modeling, sentiment analysis, e-cigarette regulation, vaping, Twitter analysis, public health related policy, marketing denial orders

## Abstract

**Background:**

e-Cigarette (electronic cigarette) use has been a public health issue in the United States. On June 23, 2022, the US Food and Drug Administration (FDA) issued marketing denial orders (MDOs) to Juul Labs Inc for all their products currently marketed in the United States. However, one day later, on June 24, 2022, a federal appeals court granted a temporary reprieve to Juul Labs that allowed it to keep its e-cigarettes on the market. As the conversation around Juul continues to evolve, it is crucial to gain insights into the sentiments and opinions expressed by individuals on social media.

**Objective:**

This study aims to conduct a comprehensive analysis of tweets before and after the ban on Juul, aiming to shed light on public perceptions and sentiments surrounding this contentious topic and to better understand the life cycle of public health–related policy on social media.

**Methods:**

Natural language processing (NLP) techniques were used, including state-of-the-art BERTopic topic modeling and sentiment analysis. A total of 6023 tweets and 22,288 replies or retweets were collected from Twitter (rebranded as X in 2023) between June 2022 and October 2022. The encoded topics were used in time-trend analysis to depict the boom-and-bust cycle. Content analyses of retweets were also performed to better understand public perceptions and sentiments about this contentious topic.

**Results:**

The attention surrounding the FDA’s ban on Juul lasted no longer than a week on Twitter. Not only the news (ie, tweets with a YouTube link that directs to the news site) related to the announcement itself, but the surrounding discussions (eg, potential consequences of this ban or block and concerns toward kids or youth health) diminished shortly after June 23, 2022, the date when the ban was officially announced. Although a short rebound was observed on July 4, 2022, which was contributed by the suspension on the following day, discussions dried out in 2 days. Out of the top 50 most retweeted tweets, we observed that, except for neutral (23/45, 51%) sentiment that broadcasted the announcement, posters responded more negatively (19/45, 42%) to the FDA’s ban.

**Conclusions:**

We observed a short life cycle for this news announcement, with a preponderance of negative sentiment toward the FDA’s ban on Juul. Policy makers could use tactics such as issuing ongoing updates and reminders about the ban, highlighting its impact on public health, and actively engaging with influential social media users who can help maintain the conversation.

## Introduction

e-Cigarette use has been a public health issue in the United States. Its prevalence increased among adults from 1.4% in 2014 to 6.4% in 2021, primarily owing to an increase among those who had never smoked cigarettes [1,2]. The upward trend was significantly higher later in the COVID-19 pandemic (April 2021-April 2022) compared with its initial months (March-July 2020) [3]. In addition, e-cigarette (electronic cigarette) use may serve as a behavioral marker of risk for mental health problems among youth, including potential depression and suicidal ideation [4,5], as well as potential pathological changes in their oral and respiratory systems [6].

Juul, which once owned a majority market share of e-cigarette sales, has garnered significant attention and controversy due to the widespread use of its products, especially among young people, and their potential health risks [7,8]. Concerns were raised by state and federal investigators, and it was determined that Juul executives knew their marketing contributed to skyrocketing youth vaping rates nationwide, reversing years of tobacco control efforts [9,10]. According to the US Food and Drug Administration (FDA), nearly 10.7 million young people from 12 to 17 years old have used e-cigarettes or are open to trying them [11]. After reviewing the company’s premarket tobacco product applications, the FDA determined that the application lacked sufficient evidence regarding the toxicological profile, raising public health concerns [12]. On June 23, 2022, the FDA issued marketing denial orders (MDOs) to Juul Labs Inc for all of their products currently marketed in the United States [12]. However, one day later, on June 24, 2022, a federal appeals court granted a temporary reprieve to Juul Labs that allowed it to keep its e-cigarettes on the market [13]. On July 5, 2022, the FDA administratively stayed the MDO. This administrative stay temporarily suspends the order during the additional review but does not rescind it [12].

From the policy maker’s point of view, as the conversation around Juul continues to evolve, it is crucial to gain insights into the sentiments and opinions expressed by individuals on social media platforms like Twitter (rebranded as X on July 23, 2023). Understanding public opinions, as well as the life cycle of an important policy announcement that moves the needle of public perceptions, can better position the government to facilitate public awareness and curb miscommunication in a resource-constrained environment.

Traditionally, the way to gather responses from the general public relies on large public surveys or small focus group discussions. It was not until recently that copious amounts of unstructured text data on social media could be processed, analyzed, and reasoned, all with the help of natural language processing (NLP) techniques, methods that integrate computer science, artificial intelligence, and computational linguistics. Over the past several years, there have been innovations in NLP research that resonate in public health and social media research [14]. More importantly, recent applications have started to adopt a cutting-edge pretraining of deep Bidirectional Transformers For Language Understanding (BERT) approach that transforms NLP tasks. NLP pipelines have been intensively developed for a variety of text-processing problems, like topic modeling, text summarization, and sentiment analysis [15]. In this study, we use these NLP techniques to delve into the social media data on e-cigarettes, allowing us to understand better the response of the general public to the evolving policy issues and the life cycle of public health-related policy concerns expressed on social media. We conducted a comprehensive analysis of tweets before and after the ban on Juul, aiming to shed light on public perceptions and sentiments surrounding this contentious topic.

## Methods

### Overview

We collected Twitter posts, responses, and retweets related to the FDA’s ban on Juul between June 2022 and October 2022. Keywords used for searching include “FDA,” “Ban,” “JUUL,” and “e-cigarettes,” and combinations of these 4. We used Twitter API v2, which has been shown to create almost complete samples of Twitter data [16]. A total of four data sets were obtained from Twitter for further processing: (1) the master file that contains Twitter post text, associated post identifier, and reply; (2) the postmetric file that contains public metrics, including the count of retweets, replies, likes, and quotes; (3) the author-metric file that contains author (tweet poster) metrics, including number of followers, following, and tweets previously posted and listed; and (4) the entity file, which indicates the potential entity that posters are affiliated with. We removed the duplicates and kept each post that had only 1 observation in the master file. We then performed one-to-one merges between the master file and the remaining 3 subsets. In total, we obtained 6023 tweets and 22,288 replies or comments and retweets.

Not only did we collect text and emoji from these posts and responses, but we also collected information regarding the date and time when posts and comments were created, as well as metrics associated with a post (ie, counts of retweets, replies, likes, and quotes) at the time of data collection. In addition, we also collected user metrics such as the number of followers a poster has, the number of accounts a poster follows, the number of tweets an author has posted from the time the account was established, and the number of publicly listed organizations of which the user is a member.

In this study, we conducted a descriptive analysis, topic modeling using the state-of-the-art BERTopic technique, and sentiment analysis.

In the descriptive analysis, we described the number of posts during the observational period to give readers an idea of how long the life cycle of “FDA’s ban on Juul” lasted. We separated the time period into 3 sections: before June 23, 2022; between June 23, 2022, and July 5, 2022; and after July 5, 2022, reflecting the fact that this ban was suspended on July 5, 2022. We counted the total number of tweets and retweets, and we recorded the selected topics over time.

For topic modeling analysis, traditionally, the field has been dominated by the Latent Dirichlet Allocation (LDA) model [17]. A limitation of this model is that, through bag-of-words representations, it disregards semantic relationships among words. As these representations do not account for the context of words in a sentence, the bag-of-words input may fail to represent documents accurately. BERTopic is an emerging topic modeling network that simplifies the topic-building process. There are various elements to model these topics. In this study, 5 procedures are processed:

1. Embedding, where documents are converted into numerical representatives. The sentence transformer used here is all-MiniLM-L6-v2, a pretrained monolingual model that compresses large transformers simply and effectively [18].
2. Dimension reduction of the input embeddings; we use the default Uniform Manifold Approximation and Projection for Dimension Reduction (UMAP) [19]. UMAP is a fuzzy topological structure-based dimension reduction technique that is used to reduce the complexity of a data set by reducing the number of features while keeping the most important properties of the original data. The default parameters were used (ie, 15 neighbors and 5 components with zero minimum distance).
3. Clustering after the dimension reduction of the input embeddings, that is, similar embeddings were clustered into groups to extract topics. The clustering is key to the accuracy of topic representations. Here, we used the HDBSCAN, a hierarchical clustering algorithm [20], to group the input embeddings into distinct clusters based on a user-specified fine-tuned cluster size (set to 15 as a default). These clusters are a rudimentary representation of potential topics. More default fine-tuning parameters were used (see the 2 repositories for more information [21,22]). The stable clusters were extracted from the condensed tree.
4. c-TF-IDF was used to get an accurate representation of the topic from the bag-of-words that were generated from the previous steps. c-TF-IDF is used, and it takes into account what makes the documents in one cluster different from documents in another cluster, and the importance score per word is calculated. The score is used to determine the presence and order within each topic that a user specifies [22].
5. In sentiment analysis, we analyzed the sentiments toward the ban (positive, negative, and neutral) of the most commonly outlined messages formulated in the tweets based on the topics generated from topic modeling. We constructed sentiment scores using VADER (Valence Aware Dictionary and Sentiment Reasoner) algorithms. VADER is a lexicon and rule-based sentiment analysis tool that is specifically attuned to sentiments expressed in social media [23]. The score is computed by summing the valence scores of each word in the lexicon, adjusted according to the grammatical and syntactical conventions that humans use when expressing or emphasizing sentiment intensity, and then normalized to be between –1 (most extreme negative) and +1 (most extreme positive) [24]. Following the literature, we set standardized thresholds to classify sentences as either positive (normalized score ≥ 0.05), neutral (–0.05 < normalized score <0.05), or negative (normalized score ≤ –0.05) [23].

We selected the top 50 most-retweeted tweets to perform content analysis and sentiment analysis. The reason we chose the top 50 is because of the skewed distribution. Of all the tweets included, those with 22 or more retweets are in the 99th percentile (mean 1.69, SD 20.34 retweets). The top 50 tweets we selected are those that were most popular, with retweet counts in the 99th percentile. Furthermore, 2 coders manually coded the theme for each post separately, with kappa ranging from 0.25 to 0.89. All the analysis was conducted in Python (version 3.6; Python Software Foundation) [25].

### Ethical Considerations

This study was exempted from institutional review board approval because data provided by Twitter academic API are publicly available and deidentified. The data was analyzed with permission from Twitter (rebranded as X).

## Results

As shown in Table 1, between June 23 and July 5, the mean (SD) retweet count was 1.47 (SD 17.85), with a higher count before June 23 (5.81, SD 46.41) and a lower count after July 05 (0.75, SD 3.17). More in-depth analysis can be found in Table 2. Similarly, the mean (SD) reply count was highest before June 23 (1.98, SD 12.07) and lowest after July 5 (0.38, SD 1.87), with a mean (SD) of 0.82 (SD 10.39) until the end of the observational period (October 22, 2022). The mean (SD) like count was 9.58 (SD 304.64) during the period of interest, with a higher count before June 23 (37.57, SD 384.17) and a lower count after July 5 (2.35, SD 11.74). Regarding author metrics, the mean (SD) number of followers was highest after July 5 (366,305, SD 2,344,692) and lowest before June 23 (143,546, SD 1,043,550), with a mean (SD) of 229,253 (SD 1,825,307) across the entire sample. The mean (SD) number of accounts followed was highest before June 23 (2707, SD 13,995) and lowest after July 5 (2061, SD 6605), with a mean (SD) of 2508 (SD 14,059) across the entire sample.

**Table 1:** Summary statistics.

			Timestamps		
		Total (N=6023), mean (SD)	Before June 23, 2022 (N=539), mean (SD)	Between June 23 and July 5, 2022 (N=4109), mean (SD)	After July 5, 2022 (N=1375), mean (SD)
**Post metrics**					
	Retweet count	1.69 (20.34)	5.81 (46.41)	1.47 (17.85)	0.75 (3.17)
	Reply count	0.82 (10.39)	1.98 (12.07)	0.82 (11.74)	0.38 (1.87)
	Quote count	0.76 (24.60)	3.92 (73.05)	0.56 (13.66)	0.12 (0.67)
	Like count	10.44 (276.78)	37.57 (384.17)	9.58 (304.64)	2.35 (11.74)
**Author matrices**					
	Author followers count	22,9253 (1,825,307)	143,546 (1,043,550)	194,634 (1,701,244)	366,305 (2,344,692)
	Author following count	2508 (14,059)	2707 (13,995)	2632 (15,793)	2061 (6605)
	Author listed count	1575 (10,057)	1115 (6412)	1314 (8877)	2534 (13,800)
	Author tweet count	141,171 (269,747)	99,475 (175,178)	139,556 (271,059)	162,345 (293,400)

**Table 2:** Content and sentiment analysis.

	Expected						Sentiment analysis^a^				Topics
Agreement, %	Agreement, %	Kappa	SE	*z*	Prob>*z*	Positive or yes		Negative or no	Neutral		
92	44.72	0.86	0.12	7.03	0	3		19	23	Whether the author supports the FDA’s^b^ ban	
80	44.6	0.64	0.12	5.25	0	2		19	21	The sentiment of Twitter post	
66	54.48	0.25	0.14	1.79	0.04	9		24	—^c^	Directly tweeted or feeds from news	
76	65.68	0.30	0.14	2.13	0.02	5		32	—	Topic: mentioning or comparing specifically about tobacco, nicotine, cigarettes	
88	81.92	0.34	0.14	2.44	0.01	2		40	—	Topic: Concerns about kids or youth	
66	52.4	0.29	0.14	2.02	0.02	11		21	—	Topic: the consequence of the FDA’s ban or block of Juul	
90	74.48	0.61	0.14	4.31	0	5		38	—	Topic: The suspension of the FDA’s ban	
88	88.	0.00	—	—	—	—		44	—	Topic: FDA’s expected move with @youtube news link	
96	63.52	0.89	0.14	6.3	0	38		12	—	With URL	

^a^Sentiment analysis numbers reflect both coders’ agreement.

^b^FDA: US Food and Drug Administration.

^c^Not applicable.

**Figure 1:**
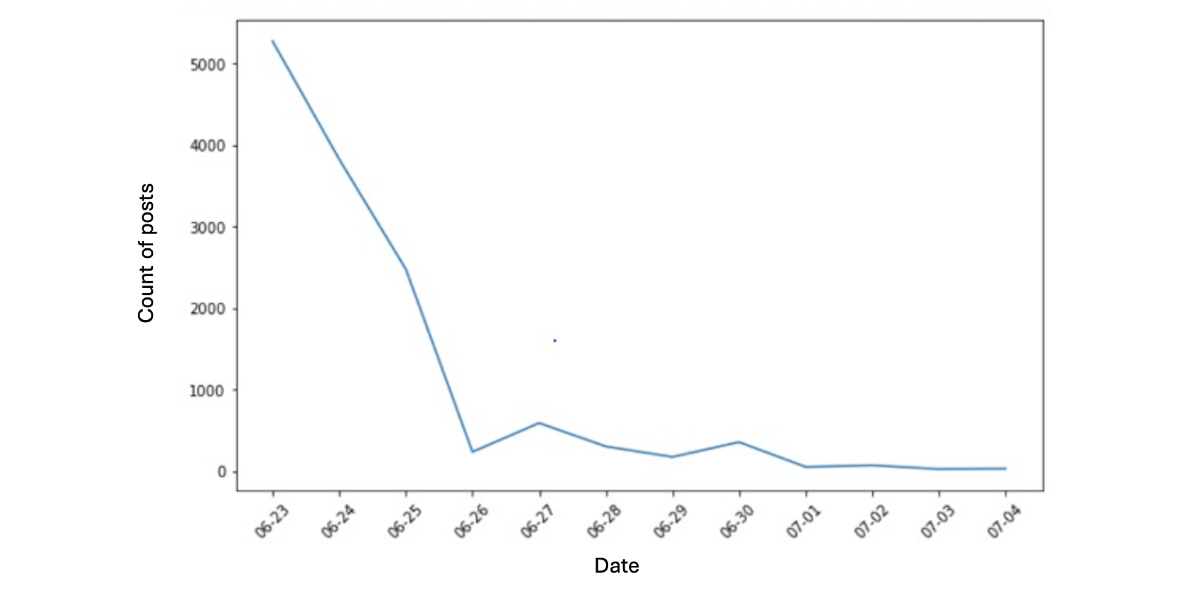
The sharp decline of tweets associated with the US Food and Drug Administration's ban.

**Figure 2:**
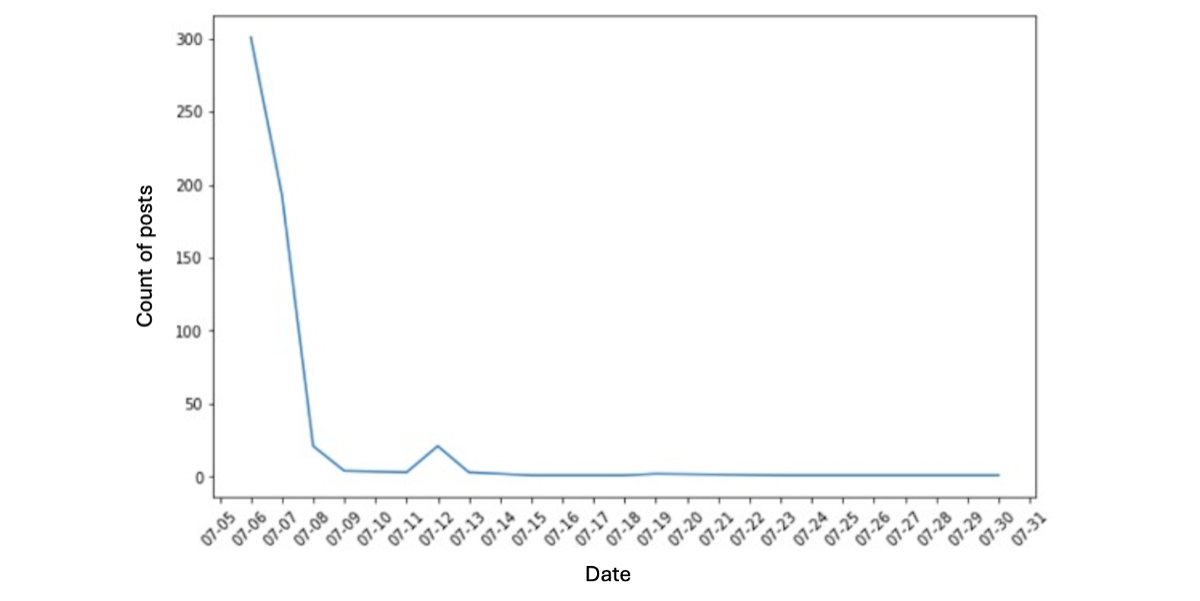
The sharp decline of tweets associated with the suspension of the US Food and Drug Administration’s ban.

Figures 1 and 2 present the time-trend analysis of all tweets related to the FDA’s ban on Juul. Figure 1 plots tweets between June 23 and July 5, 2022, the presuspension period, while Figure 2 plots the period after July 5, 2022, the postsuspension period. The number of tweets and retweets associated with the FDA’s ban experienced a significant decline between June 23 and June 26, 2022. On June 26, 2022, the quantity of posts experienced a significant decline, with only about 200 tweets compared with 5000 posts on June 23, 2022. Figure 1 shows the tweets experienced a further decline until July 5, 2022. The peak number of tweets related to the suspension of the FDA ban on Juul (Figure 2), which reached approximately 310, was observed on July 5, 2022. The number of posts dropped subsequently, down to around 25 by July 8, 2022. Tweets and retweets associated with the suspension were not observed from July 9 to July 11, 2022. A slight increase in posts was noticed on July 12, 2022, but then wound down to null until the end of the observation period.

**Figure 3:**
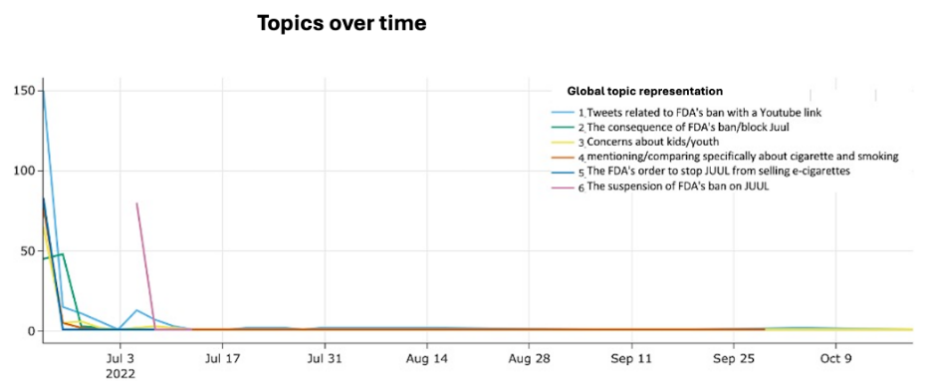
The life cycle of the top 6 topics. FDA: US Food and Drug Administration.

Figure 3 presents the life cycle of the top 6 most popular topics among the tweets related to the FDA’s ban on Juul. These 6 topics were chosen because they are most inclusive and concise (Figure S1 in Multimedia Appendix 1 for a detailed inter-topic distance map).

The topics mentioned most frequently in all collected tweets, in rank order, are (1) tweets containing the FDA’s ban on Juul with a YouTube link to a news media site or other relevant video content, as in the 2 examples below:

Prof. Peter Pitts talks about the reported upcoming FDA ban on Juul with... https://t.co/8fW2Bf31Ct via @YouTube.

**Figure 4:**
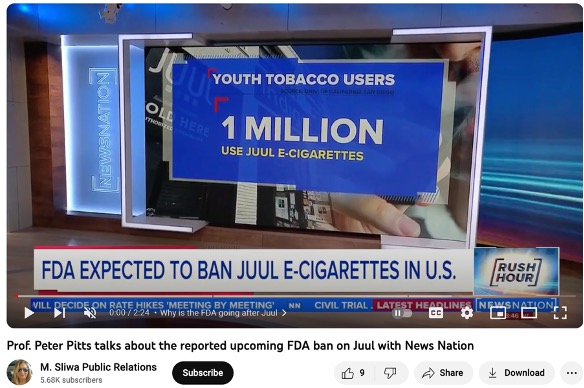
YouTube link from example post. The screenshot represents external news media sites on YouTube from tweets containing comments on the US Food and Drug Administration's ban on Juul.

The link above directs viewers to a news show talking about the FDA’s ban on Juul (Figure 4).

FDA Expected To Ban Juul Products https://t.co/m2MGiGpER2 via @YouTube.

**Figure 5:**
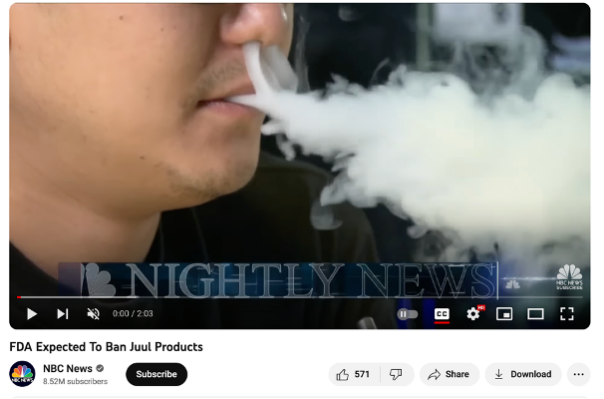
YouTube link from example post. The screenshot represents external news media sites on YouTube from Tweets containing comments on the US Food and Drug Administration's ban on Juul.

The link in this quote directs viewers to NBC News (Figure 5).

(2) Tweets talking about the consequence of a continuous ban or block on Juul, (3) tweets related to concerns about kids and youth, (4) tweets mentioning cigarettes and smoking, (5) Tweets that restated the FDA’s order to stop Juul from selling e-cigarettes, and (6) tweets associated with the suspension of the FDA’s ban on Juul. More concrete examples of word clouds are provided in Figures S2 and S3 in Multimedia Appendix 1.

We observed interesting patterns in the frequency of the 6 different topics between June 23, 2022, and October 9, 2022. Specifically, we found that Topics 1-5 experienced a peak in frequency on June 23, 2022, with a maximum of 150 tweets, followed by a sharp decline. By July 5, 2022, the frequency of topics other than the suspension of the FDA’s ban on Juul was down to near null. We observed a short rebound for Topic 1 (tweets related to the FDA’s ban with a YouTube news link) on July 4, 2022, right before the announcement of the suspension of the FDA’s ban. The rebound in the number of tweets for Topic 1 diminished quickly, followed by a peak for Topic 6 (the suspension of the FDA’s ban on Juul). The life cycle of Topic 6, however, was similar to other topics in that it faded out within a day or two*.*

Table 2 shows the result of the content analysis. In this study, we conducted a conceptual analysis, which determines the existence and frequency of concepts in Twitter text. We developed 2 sentiment concepts, 2 objective facts, and 5 topics. The 2 sentiment concepts are (1) the sentiment of Twitter posts and (2) whether the author of Twitter posts supports the FDA’s ban on e-cigarettes. The 2 objective facts are (1) whether the post is directly tweeted or feeds from news and (2) whether the post has a URL or link attached. The 5 topics are (1) related specifically to tobacco, nicotine, cigarettes, or smoking, (2) concerns of kids or youth, (3) the consequence of the FDA’s ban or block on Juul, (4) the suspension of the FDA’s ban, and (5) the FDA’s expected move with a YouTube news link. The raw agreements of these concepts ranged from 66% to 96%, with Kappa agreement statistics being significant (*P*<.05) except for 1 topic. We found, among the top 50 retweeted posts with a 2-interrater agreement of between 66% and 96%, that 23 authors (23/45, 51%) expressed a neutral opinion on the FDA’s ban on Juul (most of these retweets were redirected from the FDA’s official announcement or a news channel). A total of 19 authors (19/45, 42%) opposed the ban, and 3 (3/45, 6%) authors supported the FDA’s ban on Juul. In total, 27% (9/33) of posts were directly tweeted or retweeted from a news agency. The sentiment of Twitter posts (generated by the machine learning models), similar to the authors’ supportiveness (measured and verified by 2 independent coders), concentrated toward the neutral (21/42, 50%) and negative (19/42, 45%).

Among the 5 topics generated by topic modeling, 34% (11/32) related to the consequence of the FDA’s ban or block on Juul; 13% (5/37) mentioned or compared e-cigarettes with tobacco, nicotine, cigarettes, or smoking; 11% (5/43) talked about the suspension of the FDA’s ban; and 4% (4/42) concerned the use of e-cigarettes among kids or youth. Although many other posts among the 6000 tweets were attached to a YouTube link, none of the top 50 posts provided such a link.

## Discussion

### Principal Findings

In this study, we found that the life cycle of reactions to the FDA’s ban on Juul lasted no longer than a week on Twitter. Not only the news related to the announcement itself but the surrounding discussions (the 6 topics presented in the study) diminished shortly after June 23, 2022, the date when the ban was officially announced. Among the top 50 most-retweeted tweets, we found posters responded more negatively on the corresponding topics.

Our trend analysis findings reveal a pattern commonly observed in the life cycle of internet-based news. Our research shows a sharp increase in Twitter discussions surrounding the FDA’s ban on Juul when the announcement was made on June 23, 2022, followed by a rapid decline in 3 days. The patterns for various specific topics were very similar to the general trend. Other Twitter-based studies focusing on the FDA’s drug safety communication messaging about Zolpidem (a sedative-hypnotic used for sleeping problems) and antibiotics found that daily mention in Twitter posts spiked at the time of official announcements on these topics, reaching maximal activity within 24 hours and returning to pre-peak basal levels within 48 hours [26,27]. The sharp increase in engagement is not maintained. This pattern aligns with previous literature on the topic, which has documented the short attention span required and the fast-paced nature of internet-based discourse [28,29]. The initial surge of interest and subsequent decline can be attributed to the fleeting nature of trending topics and the constant influx of new information competing for users’ attention [30].

Although sharing a common pattern of short life cycles, differences among topics warrant a further dissection. First, concern about kids' and youths' health is one of the most popular topics associated with the FDA’s Juul ban. The discussions surrounding the topic echo the growing body of evidence showing the adverse health effects of e-cigarettes on the younger population [31,32]. Second, the potential consequence of the ban, as a categorized topic, sparked a more sustained discussion for 2 days after the announcement (Topic 2, green line shown in Figure 3).

Providing internet-based space for people to reason and debate, rather than simply preaching facts, could be a way to increase user engagement. For example, governments can partner with social media influencers that actively manage and encourage debates about important public health policies. This could not only increase the reach of information but also alter potential miscommunication on social media. Ongoing reminders about the ban could be another way to extend the duration of public engagement. Evidence showed that a reminder system on social media could enhance medical adherence to the treatment of commutable diseases [33]. We observed a short rebound of discussions about the FDA’s ban with a YouTube news link on July 4, 2022, right before the announcement of the suspension of the FDA’s ban, indicating that timely reminders may be able to prolong user engagement.

Regarding sentiment, we found that of the top 50 retweeted posts, more than half were neutral regarding the ban (where the announcement was directly retweeted or posted), while 42% (19/45) opposed the ban. We observed that Twitter users tended to be more negative toward public health policies. The distribution of sentiments shows great similarity with attitudes toward other US tobacco control laws. For example, a study focused on the Tobacco 21 law found that 42.4% (405/955) of tweets opposed Tobacco 21, and 42.6% (407/955) neither supported nor opposed the law [34]. The proportionally higher opposition is consistent with previous literature focusing on other social media sites. It has been shown that Facebook users are more likely to engage with negatively-framed antitobacco campaign posts [24]. On Twitter, research suggests that positive sentiment has dominated the discourse surrounding e-cigarettes [35]. Such positive sentiments were raised by e-cigarette advocates, along with nudging tactics, to communicate their beliefs. Positive sentiment about the use of Juul suggests that the product is being normalized among young people [36]. The positive attitude toward e-cigarettes forms a feedback loop that could be disseminated to ordinary people, making them more suspicious and more likely to hold negative opinions toward vaping-related policies [35].

In fact, similar public responses were observed in previous e-cigarette–related policies. On January 2, 2020, the FDA released the e-cigarette flavor enforcement policy to prohibit the sale of all flavored cartridge-based e-cigarettes [37]. The proportion of negative sentiment tweets about e-cigarettes significantly increased after the announcement of this FDA policy compared with before the announcement of the policy [37]. Similar negative sentiments were found when New York state banned flavored e-cigarettes [38], as well as in public response to a social media tobacco prevention campaign [39].

When looking into topics more specifically, those Twitter authors who hold negative opinions are mostly individual influencers. For example, the second, fourth, and ninth most retweeted tweets stated:

Honestly crazy as that the FDA decided to ban Juul pods before banning actual cigarettes, the agenda obviously isn’t just public health.

So the FDA wants to ban JUUL e-cigarettes for adults, but approved experimental mRNA vaccines for 6 month old infants at ~0% risk of having any complications from COVID?

The WSJ reports that the FDA will ban Juul e-cigarettes tomorrow.

Bad idea. E-cigarettes save lives.

This pattern is similar to what was found in public reactions to other e-cigarette regulations on Twitter. Lazard et al [40] found all 8 top influencers identified were actively against the FDA deeming of e-cigarettes, of which 7 of them were individual consumers and proponents, resulting in miscommunication. Miscommunication of tobacco control policy on social media sites has been documented. For example, many news tweets about the US Federal Tobacco 21 law, a sales law, incorrectly described the law as a purchase law, and some doubted its ability to limit youth access to tobacco products [41]. In addition, our selected example of the most retweeted tweets with negative sentiment showed that people often questioned the identity of the targeted groups, the intention of the authorities, and the effectiveness of the rules. This is consistent with previous research focusing on the FDA’s action to prohibit menthol. It suggested that the tweets with a negative attitude questioned the FDA’s proposed menthol cigarette rules from several angles, from the effectiveness of the rules and the targeted groups to even the feasibility of their enforcement [42].

### Empirical Contributions and Policy Implications

Our findings have important implications for policy makers aiming to prolong the life cycle of discussions and increase their reach. To extend the duration of public engagement, policy makers could use tactics such as ongoing updates and reminders about the ban, highlighting its impact on public health, and actively engaging with influential social media users who can help maintain the conversation. In addition, strategies used in other fields, such as marketing and entertainment, could be adapted to enhance the reach of the information. Leveraging storytelling techniques, creating compelling visuals, and using collaborative formats have proven effective in capturing and maintaining public attention [43,44]. Finally, using social media influencers that actively create cohesive communications could increase the reach of information and alter potential miscommunication on social media. It has been shown that the antitobacco messages had a significantly lower potential reach, received a lower proportion of impressions, and spent a lower proportion of money per message [45]. Using social influencers as message sources is a key factor for message dissemination and sustention. Evidence suggests that campaigns that used social influencers as message sources generated a greater volume of tweets per day and a broader reach per day. More importantly, the oppositional messages diminished over time, which indicates a decrease in miscommunications. Using these tactics and strategies, policy makers can aim to foster sustained engagement and maximize the impact of policy announcements in the age of rapidly evolving internet-based discourse.

### Strengths and Limitations

Although we collected comprehensive tweets to disentangle the public response to the FDA’s ban on Juul, several limitations are worth noting in this study. First, the opinions we observed might not be generalizable to the entire public since the typical Twitter users are between 25 and 34 years old [46]. Second, our analyses were limited to the data collected using prespecified keywords, which might not be exhaustively comprehensive. As a result, some public responses may not have been covered. Furthermore, the sentiment analysis did not include replies or comments to those tweets, which could result in neglecting some user sentiments. However, the number of responses was low, and the chance of underestimating positive sentiments and overestimating negative sentiments was likely minimal. Future research should develop insights into maintaining the life cycle of discussions and avoiding miscommunication. Specifically, government agencies and organizations could work with influencers to share more focused and nonjudgmental messaging about policy reasoning with fun experiences that resonate with the targeted audiences’ interests and values [47]. Researchers should also develop bottom-up agent-based simulation models to develop more insight into how tobacco control policies are disseminated and received by the general public under different social network structures.

### Conclusions

In this observational study, we found that individual Twitter users, other than regular news media, hold more negative sentiments toward the FDA’s ban on Juul. Furthermore, we observed a short life cycle for this news announcement. To extend the duration of public engagement, policy makers could use tactics such as ongoing updates and reminders about the ban, highlighting its impact on public health, and actively engaging with influential social media users who can help maintain the conversation.

## Appendix

Multimedia Appendix 1 Additional material.

## Abbreviations

BERT: Bidirectional Transformers For Language Understanding
e-Cigarette: electronic cigarette
FDA: US Food and Drug Administration
LDA: Latent Dirichlet Allocation
MDO: marketing denial order
NLP: natural language processing
UMAP: Uniform Manifold Approximation and Projection for Dimension Reduction
VADER: Valence Aware Dictionary and Sentiment Reasoner

## Data Availability

The data sets generated during and/or analyzed during this study are available in the Google Drive repository. The codes used for the analyses are available upon proper request to the authors. No generative AI was used in any portion of the manuscript writing.
