# Supplementary material for: Public Response to Federal Electronic Cigarette Regulations Analyzed Using Social Media Data Through Natural Language Processing: Topic Modeling Study
Source: J Med Internet Res. 2024 Oct 1;26:e58919. doi: 10.2196/58919 (PMC11480678; doi:10.2196/58919)
Supplement: Multimedia Appendix 1 [file jmir_v26i1e58919_app1.docx]

## Supplementary Material


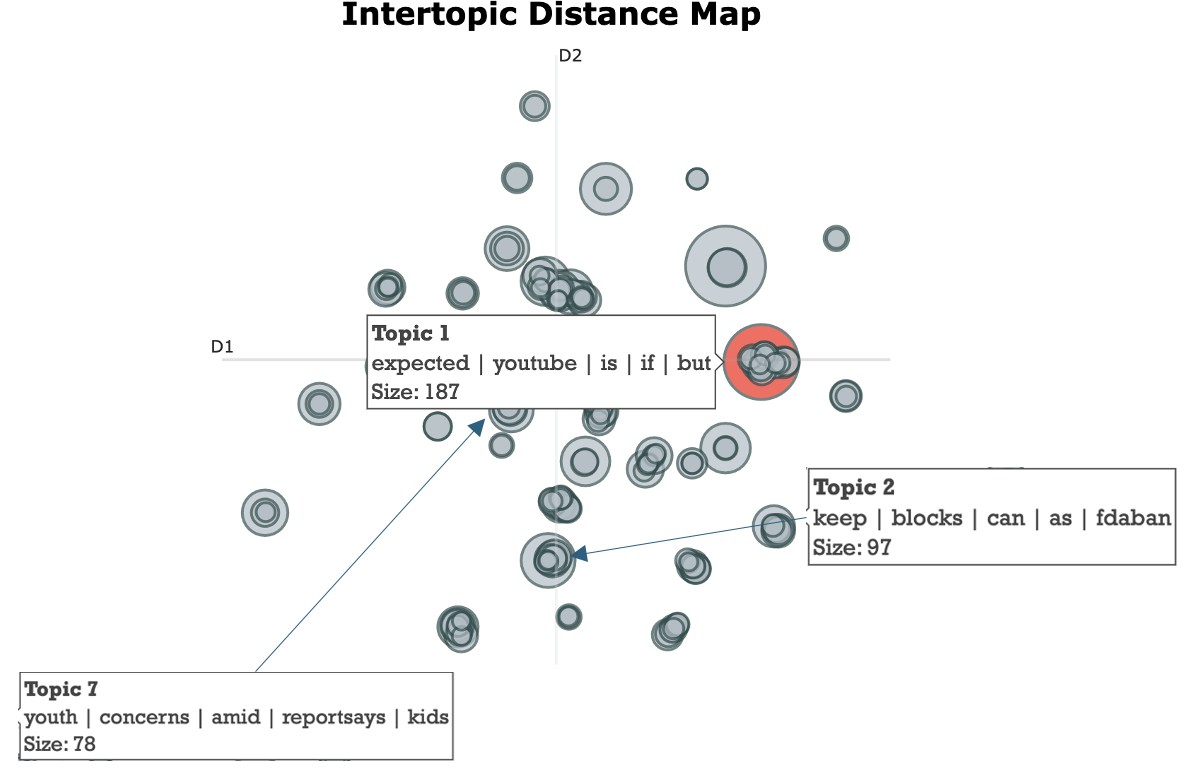


**Figure S1.** The Intertopic Distance Map. The distance map presents the all topics generated from our topic modeling. We observed many smaller circles overlapped with bigger circles, with the size of the circle indicating the number of posts categorized under that topic. Topic 1: Tweets containing the FDA’s ban on Juul with a YouTube link to a news media site or other relevant video content have 187 tweet counts. The topic with tweets related to the concerns about kids and youth incurred 78 counts.


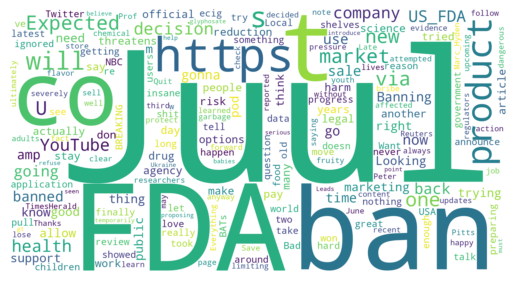


**Figure S2.** Word cloud for Topic 1: Tweets containing comment on the FDA’s ban on Juul with a YouTube link to a news media site or other relevant video content. We observed the word cloud contains several keywords that resonate with results from our topic modeling: JUUL, FDA ban, product, HTTPS, and YouTube.


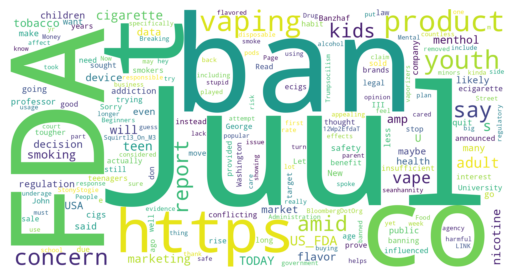


**Figure S3.** Word cloud for Topic 3: Tweets related to the concerns about kids and youth. We observed the word cloud contains several keywords that resonate with results from our topic modeling: JUUL, FDA ban, product, concern, youth, children, teen, and kids.
